# Supplementary material for: All-at-once RNA folding with 3D motif prediction framed by evolutionary information
Source: Nat Methods. 2025 Oct 3;22(10):2094–106. doi: 10.1038/s41592-025-02833-w (PMC12510878; doi:10.1038/s41592-025-02833-w)
Supplement: Supplementary file 3 — Source code to run CaCoFold-R3D. Data and results. [file 41592_2025_2833_MOESM3_ESM.gz › supplemental_material/results/CaCoFold-R3D/CaCoFold-R3D_Rfam/RF02139_HOXA11-AS1_3.R2R.sto.pdf]

RF02139_HOXA11-AS1_3

5′-Y G C C U G G U C U U Y U G G R G C Y G A G G A C U C G G G A A A C C R ● G C R R Y U G A G G C G A G C C Y ● G G G C U G C U U U A G A G G C G C U G A C A ● C C G A G G A G ● C U U C U C C U G G
